# Reasonable requests: echocardiography referral forms as a measure of coherent clinical communication

**DOI:** 10.1186/s12909-022-03602-5

**Published:** 2022-07-13

**Authors:** C. Kotzé, A. Parrish

**Affiliations:** 1 Cecilia Makiwane Hospital, East London, South Africa; 2 Department of Internal Medicine, Faculty of Health Sciences, Walter Sisulu University and Head of Department, Internal Medicine, Frere and Cecilia Makiwane Hospitals, East London, South Africa

**Keywords:** Echocardiography, Referral, Communication, Cardiac

## Abstract

**Background:**

Well performed clinical communication is a cornerstone of collaborative care in medicine but may be confounded by inconsistent intentions of the messenger and biased interpretation by the recipient. A comparison of the findings of electronic echocardiography reports with clinician-completed standardised request forms provided an opportunity to assess communication quality.

**Aim:**

The study aimed to determine clinician aptitude to complete written echocardiography referral forms by assessing the completeness, appropriateness, accuracy, and coherency of the reported clinical findings, conclusions and requests made on the referral forms. The study explored factors that may influence the quality of communication through this referral medium.

**Methods:**

A retrospective cohort study was conducted on patients who underwent trans-thoracic echocardiography imaging at Cecilia Makiwane Hospital in East London over 26 months. Paper echocardiography request forms that recorded the requesting clinician’s findings on examination, the provisional clinical diagnosis, and the specific echocardiographic information sought, were compared with the actual findings on echocardiography.

**Results:**

Of 613 request forms reviewed, 97 cases were excluded due to illegibility or because they lacked analysable information or requester details, leaving 516 forms suitable for study. No pathology was found on echocardiography in 31%. Of the murmurs expected from the echocardiography findings, only half were recorded on the request form (sensitivity and positive predictive value both 52%.). Only 35% of request forms that mentioned a mitral systolic murmur gave a working diagnosis of mitral regurgitation and only 38% of request forms that mentioned an aortic systolic murmur considered aortic stenosis. Clinically suspected cardiomyopathy (CMO) had a PPV of 43% and echocardiographic CMO was missed clinically in 41%. Apex beat displacement reported clinically was not associated with echocardiographic LV dilatation in 65% of cases. One-third (34%) of forms reporting murmurs did not request valve function assessment and 17% considering cardiomyopathy did not request left ventricular function assessment.

**Conclusion:**

Echocardiography request forms highlight vulnerabilities in clinical communication. Specifically, important clinical features were missing and more concerningly, included when unlikely to be present. There was a lack of concordance between recorded clinical findings and postulated diagnoses. Clinicians sometimes appeared unclear about the value or appropriateness of the requested assistance. Greater emphasis on teaching examination and communication skills may foster safer and more efficient use of scarce resources.

## Introduction

Efficient communication is a cornerstone of collaborative care in medicine. Specifically, requests for diagnostic assistance require a trustworthy summary of established information and a focused resolvable clinical question. Such requests meld a complex amalgam of basic history and examination skills, cognitive tasks such as accessing and ranking diagnostic possibilities and framing a question answerable by further investigation. In the team environment common in clinical practice, this process also incorporates input from colleagues (e.g. revision or re-interpretation of a physical finding), time constraints, and some level of interest and commitment to writing the request.

Although clinical communication is essential for effective, timely, and cost-effective care [1], assessing its quality is challenging. A retrospective review of communication events by audit of referral and consultation letters is vulnerable to expectation and hindsight bias [2]. An independent snapshot of the accuracy of the clinical assessment at the time of the communication event provides a more objective measure of quality than asking either the recipients of the request or observers after the event to make this judgement when they have access to more information.

Trans-thoracic echocardiography (TTE) is accessible and safe and frequently provides a definitive diagnosis [3]. TTE has been shown to correlate with intra-operative findings in up to 96.7% of cases [4, 5] with sensitivities and specificities reported up to 97% [6]. Careful portrayal of clinical findings and succinct requests to confirm or refute a specified diagnosis expedite care, but TTE requests are often perceived to be of poor quality [7]. Identifying remediable features in these requests is arguably of value to other areas of health care communication.

## Background

Clinicians receiving referrals are sometimes frustrated by their quality or brevity. Unstructured request forms allow for communication that is poorly structured, missing relevant information, or overloads the recipient with irrelevant verbiage. At our hospital, we introduced a structured echocardiography request form to address these issues [8] but found that the clinical information provided was often inadequate or inconsistent with the echocardiographic findings. This could be explained by under-developed or under-utilised clinical skills, little aptitude or inclination for filling in request forms, or even by fabrication of clinical findings, all of which have the potential to impact negatively on communication quality.

Echocardiography referral forms are a type of referral that provides objective evidence of the likely clinical findings. The structured form used in this study was intended to guide clinicians to provide clinical information in a conscientious and thoughtful manner.

Studies looking at the diagnostic accuracy of cardiac clinical examination are usually based on a once-off assessment by independent clinicians; however real-world care may mean that more than one person contributes to the clinical notes, and physical examination findings may be interpreted in the context of radiological and laboratory investigations. Echocardiography request forms are a complex amalgam of these information sources, and the communication event combines data acquisition from multiple findings, synthesis into a working differential diagnosis, and the formulation of a clinical question resolvable by the requested investigation. Missing or incorrect clinical findings suggest acquisition difficulties, discordant findings favour synthetic difficulties and missing or inappropriate reasons for the request point towards either a failure to understand the gist of the clinical problem or suboptimal focus on the task at hand.

A real-time audit may be compromised by paucity of information, and retrospective use of written replies suffers from the same potential bias. A retrospective audit is also disadvantaged by lack of access to ‘live’ clinical information which may influence interpretation, and so is usually limited to measures of completeness and coherence with little objective evidence of accuracy or appropriateness.

## Aim

The study aimed to determine clinicians’ ability to complete written echocardiography referral forms by assessing the completeness, appropriateness, accuracy, and coherency of the reported clinical findings, conclusions and requests made on the referral forms. The study explored factors that may influence the quality of communication through this referral medium.

## Methods

A retrospective cohort study covering 26 months was conducted on patients who underwent TTE imaging at Cecilia Makiwane Hospital, East London, South Africa. Structured TTE referral forms which were completed in writing and recorded the referring clinician’s findings on examination, the working diagnosis, and the cardiac parameters to be assessed on TTE, were analysed and compared to findings obtained at echocardiography.

The form prompts for basic observations (pulse rate and rhythm and blood pressure) as well as specific cardiac findings, such as the jugular venous pressure (JVP), the position of the apex beat, the presence of a parasternal impulse, the magnitude of any detected pulsus paradoxus and findings on auscultation. Apex position can be picked from a list of options, and murmurs can be filled in on a 2 × 4 grid of timing and grading [9]. The form also prompts for both a working diagnosis and specification of which echocardiographic cardiac parameters are of most interest (for instance asking about left ventricular dimensions and contractility would be appropriate in a patient with suspected mitral regurgitation.) All referral forms had to be signed by a consultant.

All echocardiography referral forms were reviewed by the technician performing the study before imaging. All echocardiographs were reported by the technician performing the study, who provided a diagnosis based on the imaging findings. The technician entered the findings into a database which generated a real-time report that also served as the source for the study information. The report provided an assessment of cardiac anatomy and physiology. The paper referral forms provided the dataset for the study. Ethics permission to perform the study was obtained from the hospital. Data collation and analysis were performed using Microsoft Access and Excel and checked in Stata 15.

## Results

In total 613 echocardiography referral forms were reviewed. Consultants filled in 117 (23%) of referrals, medical officers and registrars filled in 189 (37%), and the remaining 210 (41%) were from interns. In 69% (357 cases) pathology was found on echocardiography; the remaining 31% were reported as normal.

### Completeness and appropriateness

**Fig. 1:**
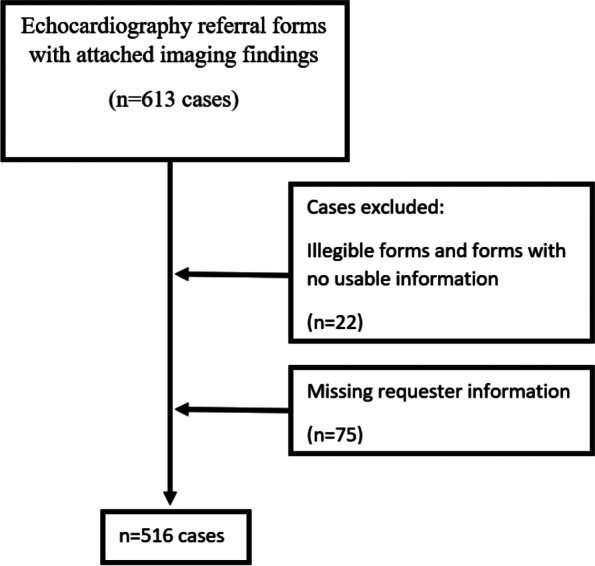
Selection of cases

Of the 613 request forms, 97 were unusable because of missing or illegible information (Fig. 1).

Ten percent of referral forms failed to provide basic clinical parameters (blood pressure, pulse rate and rhythm, JVP, apex position, and pulsus paradoxus in cases where appropriate) as prompted on the forms.

Cardiac murmurs were reported on 154 of the referral forms, and in only 102 (66%) were they accompanied by a request for assessment of valve function. In 21% of 222 clinically diagnosed cardiomyopathy cases, the clinician failed to comment on the position of the apex beat, the most accurate independent clinical predictor of LV systolic dysfunction [10].

Of the 222 clinically diagnosed CMO cases LV function assessment was not requested in 38 (17%.) However, of the 300 patients with normal echocardiographic LV function, in 132 (44%) the clinical concern was LV dysfunction. One-third (34%) of cases that reported murmurs did not request valve function to be assessed.

### Diagnostic accuracy

The referral form as a composite record of the diagnostic process performed only modestly against gold standard TTE, missing 41% of cardiomyopathies and nearly half of patients with significant valve lesions (Table 1).

**Table 1:** Performance of clinical features using echocardiography as the gold standard

	Detected clinically	Detected on echocardiography		Diagnostic performance (%)				Likelihood ratio	
Clinical feature		Yes	No	Sens	Spec	PPV	NPV	LR+	LR-
Cardiomyopathy	Yes	96	126	59	64	43	77	1.7	0.6
	No	67	227						
Valvopathy	Yes	83	74	54	80	53	80	2.6	0.6
	No	71	288						
Mitral stenosis	Yes	3	8	27	98	27	98	17.2	0.7
	No	8	497						
Mitral regurgitation	Yes	35	64	36	85	35	85	2.4	0.8
	No	61	356						
Aortic stenosis	Yes	18	53	58	89	25	97	5.3	0.5
	No	13	432						
Aortic regurgitation	Yes	4	3	8	99	57	91	13	0.9
	No	44	465						

(Trace aortic and mitral regurgitant (MR) lesions on echocardiography were classified as normal. A trace of MR was reported in 121 cases) [11].

Apex beat displacement was over-diagnosed when noted. The report of 304 patients with a displaced apex beat had only moderate sensitivity (86%), poor specificity (31%), and PPV (35%) for the prediction of LV dilatation on echocardiography.

### Internal consistency

Only 35% of cases noting apical systolic murmurs reported a clinical diagnosis of MR. The most common diagnoses when MR was not mentioned were dilated cardiomyopathy (DCMO), (21%) and CMO-unspecified (5%). Only 38% of cases that reported aortic systolic murmurs considered a clinical diagnosis of aortic stenosis (AS). The most common alternative diagnoses associated with basal systolic murmurs were DCMO, 7 cases (14%), and CMO-unspecified, 6 cases (12%). One percent of mitral systolic murmurs were erroneously diagnosed as MS. 6% of reported aortic systolic murmurs were incorrectly diagnosed as aortic regurgitation (AR).

Only 56 of the 99 mitral systolic murmurs detected on auscultation were labelled as pansystolic. 11 out of 30 cases where a tricuspid systolic murmur was detected were labelled as ejections systolic in nature.

## Discussion

At the most superficial level, the reported findings in the referral form could be considered to reflect the clinical aptitude of the referring doctor. Using the echocardiographic findings as the gold standard, it is thus feasible to construct diagnostic accuracy profiles (sensitivity, specificity, and positive and negative predictive values) for reported aspects of the physical examination in the population selected as warranting an echocardiographic assessment. These could be strictly interpreted as a surrogate of the referring clinician’s skill, but the validity of this construct is limited by the many influences impacting the written referral (Table 2).

**Table 2:** Factors influencing the quality of a referral for consultation

Diagnostic accuracy	Internal consistency	Appropriateness
Ability to elicit clinical features (clinical competence)	Ability to link findings to pathophysiology	Linkage of clinical differential to potential diagnostic gain
Technical difficulty in examination of specific patients	Tolerance of non-coherent findings	Integrity (versus inflating information to make referral appear justified.)
Ownership – personal examination rather than copying from a colleague’s notes	Time constraints and care in generating diagnosis [﻿^12^]	Impact of clinical team on personal clinical certainty (e.g., a request from a senior to complete a referral form)

Despite these reservations, the referral forms are a pragmatic reality of hospital practice and represent the interface between the echocardiography service and individual clinicians or clinician teams. Their quality is thus a valid target for audit and potential improvement.

### Completeness of forms

Missing information is an important vulnerability in medical communication, prompting the development of checklists and formalised handover structures such as SBAR (Situation, Background, Assessment, Recommendation) [13] which have gained popularity as mechanisms for addressing this. The echocardiography referral forms allow for concise yet complete portrayal of relevant clinical findings. The form aims to guide clinicians in their assessments. Despite this, 16% of forms were omitted from the study due to missing essential information.. From the forms included in the study, an average of 10% of basic patient information was omitted.

Clinicians at our centre are aware that all patients will receive a comprehensive TTE study regardless of the content of the referral form. All cases, even those excluded from the study, received echocardiographs. The comprehensive completion of the referral form may be seen only as a step in justifying the requested study, and the clinician may be unaware of, or indifferent to, the requirement that imaging findings should be correlated with clinical findings in order to make an accurate, combined diagnosis. Potential contributing factors include an overburdened health care system, underdeveloped or underutilized clinical skills, clinician indifference or ignorance, and tolerance for poorly completed referral forms. The practice of having consultants sign every request adds to workload, especially if they review every case comprehensively. This may have led to consultants signing referral forms without review, resulting in failure of this quality control.

Of particular interest was the potential generation of false-positive clinical findings. There were 99 mitral systolic murmurs detected on auscultation but 64 of these (65%) were not confirmed on echocardiography. Similarly, 57% of reported tricuspid murmurs could not be confirmed on echocardiography. Displacement of the apex position was also over-reported in 65%. These finding may reflect a desire by the clinician completing the form to generate a valid reason for the referral but raises concerns about tolerance for over-calling or even fabricating findings as a way to justify referrals. These findings also highlight the vulnerability of written referral forms to confirmation and expectation biases.

### Performance of the echocardiography referral form as a clinical diagnostic tool

The referral form, acting as a composite measure of bedside clinical diagnosis, performed poorly. On the one hand, the clinical examination can be challenging (patient factors), but conversely, the diagnostic performance may represent difficulties clinicians experience in eliciting standard clinical findings (clinician factors).

Multiple factors may contribute to valve lesions being missed clinically. The most direct explanation is that clinicians have not developed the required skills to detect these lesions. Gardezi et al. reported similar results to these, showing that general practitioner auscultation of asymptomatic patients with mild valve lesions had a sensitivity and specificity of 32 and 67% [14]. Clinicians are possibly not examining patients completely before requesting echocardiographs, resulting in incomplete findings. The low clinical sensitivity to predict valve lesions is most likely a combination of all the above-mentioned factors. Robert et al. reported similar findings with clinical auscultation by doctors having 38 and 75% sensitivity and specificity for detecting rheumatic heart lesions in high-risk populations [15].

O’Neil et al. also reported a low PPV of 59% for a displaced apex beat to predict cardiomegaly as confirmed on basic radiography [16]. This may reveal shortcomings in the skill of clinicians to assess the position of the apex beat. More likely, clinicians exhibit expectation and confirmation biases when performing their examinations, as they expect their clinical findings to confirm the clinical suspicion already reached.

### Internal consistency

This reflects the writer’s gist understanding of the suspected pathology and the ability to turn it into a coherent diagnosis.

The character of mitral systolic murmurs was not described at all or was labelled as ejection in 43%, contrary to the higher probability that an isolated apical systolic murmur is pansystolic. In only 1 of the 13 cases described as ejection systolic was AS detected on echocardiography, possibly implicating a Gallavardin phenomenon, however, these imaging findings suggest that no ejection systolic murmurs would be expected over the mitral region in the remaining 12 cases. Similarly, 11 of 30 reported tricuspid systolic murmurs were concerningly reported as ejection systolic in nature.

In 62% of referrals where an aortic systolic murmur was heard, AS was not mentioned as a key concern when requesting diagnostic assistance from echocardiography. Several aortic systolic murmurs were labelled as due to aortic regurgitation. These internal inconsistencies may reflect either gaps in understanding of cardiac examination or lapses of attention when collating information.

Patients with valvopathy on echocardiography were often only assessed as cardiomyopathy. These findings suggest a “something is wrong with the heart” approach as opposed to relying on the clinical examination to reveal the underlying pathology.

### Appropriateness

Directed requests for the measurement of echocardiographic parameters likely to guide clinical management reflects the ability to understand the utility of the echocardiography referral.

In nearly one-third (31%) no cardiac pathology was detected on echocardiography, with 69% of referrals in this group being to assess LV function. This raises interesting issues around the safety net provided by this type of service. Even highly experienced clinicians may struggle to safely exclude LV dysfunction in patients with a compatible history and a challenging physical examination (e.g., due to severe obesity.) Whether one-third of echocardiographic examinations proving negative is appropriate is situation-dependent; in environments with lower levels of cardiac examination skills it may be acceptable but in environments with ready access to adequate clinical examination skills a lower rate may be appropriate.

In 17% of clinically detected cardiomyopathy patients, there was no request for assessment of left ventricular function, and in 34% with detected valvopathy, valve function was not requested. Explanations for this are speculative but may reflect a lack of conviction in the diagnosis reached, an assumption that the echocardiographer could proceed without the need to provide direction, or possibly a lack of engagement with the referral form as a communication tool.

## Limitations of the study

Retrospective observational studies are limited by the fixed nature of the available information, and prone to confounding by unmeasured variables. Some examples are those affecting ease of examination (e.g., obesity) and the complex calculus around determining how referring clinicians filling in the form perceived their role. Clearly those less enthused when describing their cardiac examination findings and constructing a diagnosis and investigative strategy, and those more inclined to see the form as a necessary chore, would bias results towards poorer performance.

## Study strengths

The dataset was large enough to provide some conclusions about this aspect of communication and is a pragmatic study reflecting real-world practice, giving insights into actual clinical behaviour. Finally, the echocardiographic findings obtained at about the same time as referral generation provided an objective measure against which to judge referral quality.

## Conclusion

Clinicians communicate poorly through written referral forms. The referral form, acting as a composite measure of bedside clinical diagnosis in a non-simulated clinical milieu, performed poorly. Specifically, important clinical features were missing and more concerningly, included when unlikely to be present. There was a lack of concordance between recorded clinical findings and postulated diagnoses. Clinicians sometimes appeared unclear about the value or appropriateness of the requested assistance.

Echocardiography referral forms highlight vulnerabilities in written clinical referral systems. We identified system factors (strained human resources, tolerance for poor referrals) and clinician factors (poorly developed or under-utilized clinical skills and insight, ignorance/indifference to clinician roles in referral systems, bias) that contributed towards poor referral letters.

## Future areas of focus

There is potential for improvement both in training to elicit cardiac findings as well as in fostering the clinical insight to synthesize findings into a logical clinical diagnosis. Even with a form guiding data entry, accuracy, completeness and coherence could all be improved and warrant closer attention during undergraduate training.

Electronic referral systems may allow for improvement in quality and comprehensiveness of referrals as these systems can include internal quality control checks. Electronic referral systems have been shown to reduce missing information and improve quality of referrals and have been shown to generally improve clinician satisfaction [17].

Feedback on the quality of referral letters and greater emphasis on teaching examination and communication skills emphasising the responsibilities of different role players in the communication system and the need to construct and convey coherent and accurate data, may foster safer and more efficient use of resources [18].

Areas of interest for further research include use of electronic systems with built in quality control prompts, and the use of interview techniques to investigate how clinicians perceive the underlying communication process involved in requesting specialised investigations of this nature. Another area of considerable interest would be whether prompt-based data entry leads to provision of some less accurate information, and how clinicians perceive that potential clinical communication hazard.

## Data Availability

The datasets used and/or analysed during the current study are available from the corresponding author on reasonable request.

## Abbreviations

AR: Aortic regurgitation
AS: Aortic stenosis
CMO: Cardiomyopathy
DCMO: Dilated cardiomyopathy
JVP: Jugular venous pressure
TTE: Trans thoracic echocardiography
LR+: Positive likelihood ratio.
LR-: Negative likelihood ratio
LV: Left ventricular
MR: Mitral regurgitation
NPV: Negative predictive value
PPV: Positive predictive value
Sens: Sensitivity
Spec: Specificity
